# Valence and Origin of Emotional Words Influence on Subsequent Perception of Ambiguous Stimuli in Terms of Competence Versus Warmth

**DOI:** 10.1007/s10936-017-9505-z

**Published:** 2017-06-21

**Authors:** Kamil K. Imbir

**Affiliations:** Faculty of Psychology, University of Warsaw, 5/7 Stawki St., 00-183 Warsaw, Poland

**Keywords:** Social cognition, Ambiguous stimuli interpretation, Priming, Dual-processes theories of mind, Origins of emotions

## Abstract

The aim of this study was to examine whether the valence and origin of emotional words can alter perception of ambiguous objects in terms of warmth versus competence, fundamental dimensions of social cognition. 60 individuals were invited into the study focusing on the limits of intuition. They were asked to try to guess the meaning of Japanese pictorial signs in terms of their warmth versus competence connotations. Before each trial a subsequent memory load task was applied. Participants were supposed to read and remember words creating a factorial manipulation of valence (three levels) and origins (three levels: automatic, neutral and reflective) of affective connotations presenting to them for 500 ms. For positively valenced words, automatic originated ones resulted in perception of ambiguous signs more in terms of warmth, while reflective originated words resulted in perception of signs more in terms of competence. This study shows that social perception of warmth versus competence is susceptible to emotional influence of unrelated stimulation, and thus can be primed by objects in the environment. Warmth may be treated as linked with automatic mind processes, while competence may be treated as associated with the controlled part of the mind. In a broader context, this experiment results support claim that distinct dualities identified in dual-processes theories of mind are related to one another, and in fact they may be emanations of two more general systems of mind.

## Introduction

The affective meaning of external stimuli is thought to influence the state of mind of individuals. If one cries while reading a sad story, the question can be asked: how many different aspects of the reader’s state of mind are affected? Especially, would incidental affect alter the use of dimensions of social perception in understanding an ambiguous stimuli? In this paper the effects of affective reactions to verbal stimuli (presented as unrelated task, thus inducting an incidental affect) on interpretation of ambiguous stimuli related to social environment are examined. Both affective reactions to stimuli (Imbir 2015) and social cognition (Abele and Wojciszke 2014; Fiske et al. 2007; Wojciszke 2005) are defined from perspective of dual-processes theories of mind (Gawronski and Creighton 2013). In emotional domain the distinction between so called automatic versus reflective originated affective reactions to verbal stimuli is considered (c.f. Imbir 2016a, b; Jarymowicz and Imbir 2015). In social cognition domain the interpretation of social related stimuli in terms of two fundamental dimensions of warmth and competence is tested (Fiske et al. 2007).

### Dual Mechanisms of Social Perception

Social perception is one of the most important issues concerning modern and historical societies. Fiske et al. (2007) argue that warmth and competence are the crucial dimensions in social perception and social cognition (cf. Wojciszke 2005). Warmth can be connoted by labels including generosity, helpfulness, honesty, righteousness, sincerity, tolerance, and understanding; while competence is exemplified by other labels, including cleverness, creativity, efficiency, foresight, ingenuity, intelligence, and knowledgeability (Fiske et al. 2007; Wojciszke 2005). Numerous studies have shown that warmth and competence account for the majority of variance in social perception, including approach-avoidance tendencies (Cacioppo et al. 1997; Peeters 2002) or inference about the motives of other people (Reeder et al. 2002). It seems that warmth judgements are primary in terms of time, in that they appear before competence judgments, and they are more salient than competence judgments in guiding behavior (Fiske et al. 2007). Warmth is therefore most likely the earlier dimension from an evolutionary perspective (Imbir 2017a). Put simply, when an unknown person is approaching us, it is more important to get to know their intentions (good or bad) than get to know their abilities (low or high) to realize those intentions. In lexical decision tasks, warmth-related words are discriminated from pseudo-words faster than competence-related words (Ybarra et al. 2001). Also, a 100 ms exposition of faces resulted in more reliable assessments on warmth (trustworthiness) than competence dimensions (Willis and Todorov 2006).

The prioritization of the warmth dimension can be related to the dual-process framework (cf. Gawronski and Creighton 2013; Kahneman 2011) differing between two types of processes: automated and controlled ones. Each of these processes originated in separate mind systems (cf. Epstein 2003; Kahneman 2003, 2011) and is based on distinct cognitive processes (c.g. Strack and Deutsch 2004, 2014). Automated processing is the default mode of the human mind (Kahneman 2011) based on heuristics and other cognitive shortcuts. It appears to work effortlessly and very quickly gives an approximation of an answer, which in most of cases is good enough to get by with. Controlled processing is based on systematic thinking (Kahneman 2003), uses algorithms, rules of logic (Epstein 2003) and propositional mechanisms based on the operation of meaning (Strack and Deutsch 2004). For that reason, controlled processing takes more time and is less frequently activated in everyday situations. Only when motivational aspects of stimulation are strong enough, is controlled processing activated, giving, almost in all cases, the correct answer (Imbir 2017a).

The data concerning warmth and competence time differences may be interpreted as the results of processing in the experiential versus rational mind (Epstein 2003). Also, the meaning and associations of warmth and competence suggest that both dimensions are emanations of two mind systems.

### Emotion and Cognition: Duality of Mind Perspective

The duality of mind perspective offers an intriguing insight into mind processing (cf. Gawronski and Creighton 2013), but at the same time is an object of criticism because of the large number of dualities investigated in this approach (Keren and Schul 2009). The aim of the current study is to focus on similarities rather than differences between dual-mind approaches. I would like to stress that the most important contribution of dual-mind theories are the mechanisms proposed for processing (automated, effortless, and experiential, vs. controlled, effortful, and rational). Those mechanisms can be found in cognitive as well as emotional processes (Imbir 2016a). In cognitive psychology, the status of dual-mind theories is well established (Gawronski and Creighton 2013), but in the emotional domain, the dual nature of affective processes has not been the subject of special attention. For example, Kahneman (2011) or Epstein (2003) treat emotions as part of a single mind system responsible for heuristic thinking as well as experience. In fact, the possibility of reflective evaluations was proposed in the Strack and Deutch model (2014). Their nature was described as based on verbal criteria of evaluation (cf. Reykowski 1989) to which certain situations are compared. The same mechanisms can be found in the Jarymowicz and Imbir (2015) model.

The main assumption is that the origins—either automatic or reflective—of each mental system’s emotional representation provide a base for emotional states (Jarymowicz and Imbir 2015). Automatic originated emotions are those based on non-verbalized criteria of evaluation such as biological value, as postulated by Damasio (2010). This criterion is a universal one, developed due to evolutionary processes. To put it simply, everything that helps maintain life is associated with pleasure emotions, while everything that threatens survival is associated with displeasure. Such emotions are effortless and appear immediately after environmental stimulation. For example, when one is watching another person’s face, it is extremely easy for most healthy individuals to perceive the emotions expressed on this face (Kahneman 2011). Certain expressions are associated with certain probable behavior; thus, an angry face is easy to understand and individuals can infer possible outcomes when facing an angry man. Although automatic emotions do not require the presence of language (Jarymowicz and Imbir 2015), they are represented in language as labels of automatic originated states (e.g. anger, pain etc.).

Reflective originated emotions are those based on verbalized criteria of evaluation (cf. Reykowski 1989) and appear due to mechanisms comparisons between actual state and the criterion of evaluation (Strack and Deutsch 2014). Reflective emotions can be related to self-conscious emotions (Lewis 1995; Weiner 2005) or feelings (Damasio 2010). Language is crucial to evoke these emotions, because verbalization makes it possible for propositional mechanisms to work (Strack and Deutsch 2014). The processing of such emotions is effortful, because it involves a lot of mental operations, including the creation of situation representation, creation of active standard representation, and comparison between both. In these types of emotional processes there are no fixed patterns as can be seen in the case of automatic originated emotions. Every situation can be interpreted in a different way by different people; an individual may interpret a single situation as eliciting different reflective emotions, depending on the active evaluative standard created.

In order to avoid the methodological problem that arises with different contents of automatic and reflective emotional processes, which may elicit discussion, the approach proposed here focuses on stimuli associated with two previously described mechanisms of emotion formation. To this end, a Self-Assessment Manikin (SAM) scale for two mind systems was created (Imbir 2015) and a huge number of words were checked for their affective connotations concerning origins and other dimensions like valence, arousal, or concreteness (Imbir 2015, 2016b). The SAM origin scale is based on a well-known heart versus mind dichotomy, representing processes associated with passionate, irresistible, and hot matters of heart (metaphors for automatic processes and experiential mind) on one hand and calm, sophisticated, and cold calculations of the mind (metaphors for reflective processes and rational mind) on the other. It seems that the origin scale allows for reliable measures of perception of one’s affective reaction to stimuli (Imbir 2015). On the basis of those ratings, the current experiment was created (cf. methodology section). This brings us to the most important question concerning the current work: is perception in terms of warmth versus competence susceptible to the emotional quality of environmental stimulation?

### Proposed Theoretical Model

When considering the emotional quality of stimulation, the most intuitive aspect of it is emotional valence (Kagan 2007). In evolutionary terms, valence represents the intuition of perceived chances to survive or achieve goals or expectations (Tooby and Cosmides 1990). Negative valence is associated with objects or situations that decree fitness (Damasio 2010) or different kinds of threat to life. Such situations demand effort from an organism to change the current state into something more bearable, thus, there is no space for simplified processing that may lead to errors. Alternatively, positive valence is a signal that everything is going well and organisms don’t need to change their behavioral strategies. Positive mood therefore promotes cognitive laziness (Kahneman 2011) and simplified cognition including the use of some kind of stereotypical knowledge and expectations towards safe environments. The dimensions of warmth and competence are to some extent part of stereotypical social knowledge (Fiske et al. 2007; Wojciszke 2005), meaning that they allow the labelling of social categories and the prediction of possible future interactions with social objects. Taking this into account, one may expect that when searching for outcomes of emotional stimuli in the perceptual dimensions of warmth and competence, the results may be found only in the condition of positive valence, allowing the use of a simplified mode of social reasoning.

**Fig. 1:**
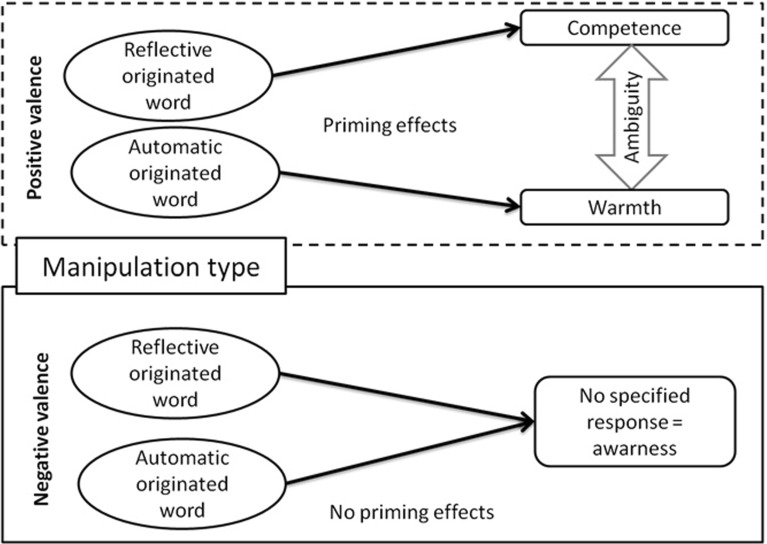
The theoretical model presenting valence and origin of words effects on warmth versus competence perception of ambiguous Japanese signs

Earlier studies into the dimension of warmth (Ybarra et al. 2001; Willis and Todorov 2006) suggest its association with the automatic mind system and therefore automatically originated positive-valenced stimuli should promote social perception in terms of warmth rather than competence. An opposite pattern of relationship should be observed when stimuli that are reflective in nature are considered. More effortful processing of reflective originated and positive valenced stimuli should promote social perception in terms of competence rather than warmth. The described theoretical model is presented in Fig. 1.

This theoretical model raises another question. What type of task would best examine the relationships described above? In psychology there is one important type of task that is susceptible to manipulations of state of mind, allowing the measurement of subtle outcomes of mind processes. This is an ambiguous task.

### Ambiguity as a Method for Assessing Proposed Model

Ambiguity can be defined as the state of having more than one possible meaning or having more than one possible answer, especially when a considered stimulus has no particular meaning to the person (Mendes et al. 2008). Ambiguity accompanies our lives constantly: we face it in almost every sphere of functioning. Mostly, it is related to human behavior and manifests itself in elements of personality such as sense of humor or irony. Ambiguity also enables us to make art and explain some abstract concepts (notions and states that are not tangible and which can be expressed by metaphors). It seems that ambiguous situations should be perceived positively, because they create an area to interpret reality in terms of our own beliefs and preferences.

However, what if we are asked to deal with a task in which the choice is unclear? Generally, people do not feel comfortable when deciding under uncertainty. According to the Ellsberg Paradox (Ellsberg 1961), we usually choose the option with the lesser amount of ambiguous content. So-called ambiguity aversion is critical to the competence hypothesis (Heath and Tversky 1991). This establishes that choices involving areas of our knowledge are preferred. If the situation is ambiguous, we would rather make a choice, provided that there is no big difference between what we know and what can be known. Thus, people are not willing to make decisions under uncertainty; however, if there is no other way, having knowledge concerning the area of choice is desirable.

What happens when we face an ambiguous dilemma and no knowledge is available? Studies on ambivalent attitudes give us interesting background information. Ambivalence refers to conflicting points of view that produce contradictory emotions and make the choice difficult. For example, van Harreveld et al. (2012) emphasize that, although generally there is a need to invest time and consider all the pros and cons when deciding on ambivalence, if evidence is limited, a strategy of minimizing cognitive effort dominates. Additionally, Zemborain and Johar (2007) revealed that for individuals solving highly ambivalent tasks, reliability of information is less important for participants facing weak ambivalence. It indicates that heuristic processing is an efficient way of reducing conflict if the decision is unclear. High motivation to make a choice is associated with lower attentiveness to evidence, as there is a will to solve the task quickly and stave off the uncertainty.

For that reason, ambiguity is a useful tool for researching consequences of subtle manipulations in psychology. For example, Murphy and Zajonc (1993) developed an affective priming paradigm, in which the participants’ task was to judge signs that were unfamiliar to them, taken from the Chariness alphabet. Each probe was primed (optimally or suboptimally) to show faces expressing positive or negative emotions. In some of their experiments, participants were told that the presented stimuli represented objects that could be bad or good. This was, in fact, an ambiguous task, because participants had no rational clue how to answer the question. Using an affective priming paradigm, Blaszczak and Imbir (2012) developed an ambiguous task, allowing the measurement of implicit self-reference effect with the use of a hexagram as meaningless stimuli that were used to judge how to describe traits connected with oneself. Hexagrams depicted stimuli on vertical white and dotted black lines. In this experiment, hexagrams were suboptimally primed by faces expressing emotions. The task was to infer to what extent a trait represented by a hexagram in the far-east pictorial alphabet is applicable for describing oneself. This ambiguous task identified that responses made by participants were sensitive to the subliminally presented facial expressions of positive and negative emotions. Another paradigm designed to measure the effects of incidental affect with use of Chinese Ideogram signs, introduced as Affect Misattribution Procedure (Payne et al. 2005), showed that even political attitudes can be the sources of affect derived from pictures of candidate one is intended to vote for in presidential election.

Tasks similar to the all described above could be useful for detecting subtle affective manipulations of a state of mind, since ambiguity is also present in social cognition. When we try to categorize a person that we do not know, we can use either available contexts or motivational expectations if the environmental cues or our knowledge are not sufficient (Pauker et al. 2010). In the latter case, initial impressions have great importance and individuals perceived at first sight as in-group members are favored. In the Rule et al. (2008) study concerning men’s sexual orientation, non-obvious, ambiguous features (subtle areas such as the eyes and mouth) were used to make intuitive judgments. It is highly possible that in the case of unknown objects representing indefinite categories, we also rely on first impressions and quickly act in order to diminish perceptual ambiguity. For that reason, the properties of ambiguous task materials are important and need to be considered carefully in order to minimize the tendencies described above.

### Aim and Hypothesis

The aim of the current work was to establish whether perception—as defined in terms of the warmth and competence—of stimuli suggested to be a symbolic representation of personality traits in far-east pictorial alphabet is susceptible to affective state manipulation. The affective manipulation was designed as caused by an unrelated to the main task presentation of words that. The affective manipulation was operationalized with regard to affective connotations of words. They were measured using two different Self-Assessment Manikin (SAM) scales designed for assessments of valence (Lang 1980) and origin of an affective reaction (Imbir 2016b). Valence varied from negative to positive with neutral as control conditions. Origin SAM was developed by Imbir (2015) in order to measure subjective perception of own affective reaction as caused by automatic versus reflective mechanisms of evaluation (Jarymowicz and Imbir 2015). To measure origin a heart versus mind metaphor was used, exemplifying two modes of processing: (1) automatic, in other words overwhelmed with appeals from the heart, including experiences of being beside oneself, complete commitment, full engagement, impulsivity, spontaneity or lack of hesitation and (2) reflective, in other words under the sway of the mind, including feelings that result from contemplation, planning, consideration, prediction, choices, or comparisons. As stated, origin varied from automatic (“dominated by a reasons of heart”) to reflective (“dominated by a reasons of mind”) with control condition based on using a words of non-specified origin, meaning they were neither reflective in origin nor automatic in origin.

In the case of negative valenced stimuli, there was no expectation to find differences due to the fact that negative valence gives information about danger, thus promoting careful consideration. For that reason, the perception of unknown objects should not be primed in any direction (cf. Fig. 1). In other words, potentially dangerous objects should be inspected carefully, thus there are no useful expectations.

In the case of positive valenced stimuli, the effects of origin were expected. Automatic originated positive words should enhance warmth perception, while the reflective originated stimuli should promote competence responses. This expectation is based on the assumption that warmth is associated with the experiential mind system, thus with automatic originated emotions; while competence is associated with the rational mind system and thus with reflective originated emotions. Such expectation is also consistent with the claim that Affect Misattribution Procedure (Payne et al. 2005) may be more sensitive to meaning or semantic aspects of priming stimuli, rather than affect defined as valence itself (Blaison et al. 2012). The expected origin effect to some extend is an example of semantic congruency in terms of complexity of underlying mechanisms congruency. No specific predictions were established concerning reaction latencies.

## Method

### Participants

The experiment involved 60 students (30 women and 30 men) from different universities. They represented a variety of faculties (biology, technology, linguistics, economics, law, etc.) and participated in the study in exchange for gift cards. The participation was voluntary. Participants were aged from 19 to 27 years, with a mean age of 21.48 years (*SD* $=$ 2.12 years). The age range in the current experiment was chosen in order to be congruent with the age range of the sample assessing verbal stimuli used to create factorial manipulation (Imbir 2016b). The sample size was determined in advance as 60 participants and data collection was terminated when the sample size reached the quota. All participants had normal or corrected-to-normal vision. Only Polish language native speakers with no knowledge of far-east languages were invited to participate in the study. The experimental protocol was approved by an institutional ethical review board of Maria Grzegorzewska Academy of Special Education and study was carried out in accordance with the provisions of the World Medical Association Declaration of Helsinki.

### Linguistic Materials

To manipulate the valence and origin of the affective state in individuals, words were chosen as research materials. This choice was justified by the nature of the verbal materials: they expressed all objects or states in human world (Imbir 2015), both from automatic and reflective origins. The linguistic materials chosen consisted only of nouns divided into nine groups of 15 words each. All groups were controlled for aligned levels of arousal, concreteness, length, and frequency of appearance, but they differed in levels of valence and origin so as to form a 3 (valence) $\times$ 3 (origin) factorial manipulation.

The selection of words was based on a normative study using 4905 Polish words, for which valence, origin, arousal, and concreteness were assessed by at least 50 students (25 male and 25 female) from different universities (“Appendix”, also: Imbir 2016b). They were students of different faculties, and the pool was well balanced. The whole study (Imbir 2016b) involved 400 participants (200 females) aged from 18 to 32 ($M = 21.89$, *SD* $=$ 1.91), students from different Warsaw universities and colleges of social sciences (excluding psychology students) and humanities (36%, $N = 144$), natural sciences (32%, $N = 128$), and technical sciences (32%, $N = 128$). The proportion of sexes across faculty types was balanced (50% female in each case) in order to avoid any sex bias over affective evaluations. The task for the participants was to evaluate a list of 4920 words (15 were doubled in order to provide additional estimation of reliability (c.f. Imbir 2016b)) using a single SAM scale described in detail at the beginning of procedure. The study was done using the same methodology as in the previous normative study consisting of 1586 Polish words (Imbir 2015).

To achieve different levels of valence and origin, words were selected and rated respectively: below −1 *SD* (negative valence or automatic origin), from −0.5 to 0.5 *SD* (control conditions of both dimensions), and above 1 *SD* (positive valence or reflective origin) from the average rating in the corresponding dimension. Further, the selected words had medium ratings (between −0.5 and 0.5 *SD*) for arousal and for concreteness.

The selection procedure also ensured an equalization of the frequency of appearance and length (Number of Letters: NoL) of words. Frequency estimations were based on online internet Polish texts (Kazojć 2011) and represented the number of occurrences of each word in the whole database used. The distribution of values in this database was right-skewed, but was corrected by natural logarithm (ln) transformation, enabling the application of parametric statistics. Thus, all analyses were conducted with use of the ln transformations of frequency estimation. This list was prepared for an earlier study involving the valence and origin of emotional meaning of words in their processing (Imbir et al. 2016).

To ensure the correct construction of the manipulation, we conducted 3 (valence levels) $\times$ 3 (origin levels) ANOVA analyses for each dimension measured. Table 1 presents the pattern of obtained results. Table 2 presents the mean values (*M*) and standard deviations (*SD*) of assessments for each manipulated group of words. “Appendix” presents all words used and their normative values.

**Table 1:** Properties of the experimental words

Dimension	Main effect of valence words groups	Main effect of origin words groups	Interaction of valence and origin words groups
Valence	${{\varvec{F}}}\mathbf{(2,126) } = \mathbf{607 }.\mathbf{44 },\,{{\varvec{p}}}< \mathbf{0.001 }, {\varvec{\eta }}^{2} = \mathbf{0.91 }$	*F*(2,126) = 1.88, *p* = 0.16, $\eta ^{2} = 0.03$	*F*(4,126) = 2.09, *p* = 0.086, $\eta ^{2} = 0.062$
Origin	*F*(2,126) = 1.27, *p* = 0.28, $\eta ^{2} = 0.02$	${{\varvec{F}}}{} \mathbf (2{,}126) = \mathbf{254.55 }$, ${{\varvec{p}}}< \mathbf{0.001 }$, ${\varvec{\eta }}^{2} = \mathbf{0.80 }$	*F*(4,126) = 0.5, *p* = 0.74, $\eta ^{2} = 0.016$
Arousal	*F*(2,126) = 1.98, *p* = 0.14, $\eta ^{2} = 0.02$	*F*(2,126) = 1.44, *p* = 0.24, $\eta ^{2} = 0.02$	*F*(4,126) = 0.5, *p* = 0.72, $\eta ^{2} = 0.016$
Concreteness	*F*(2,126) = 1.19, *p* = 0.31, $\eta ^{2}= 0.02$	F(2,126) = 0.4, *p* = 0.67, $\eta ^{2} = 0.006$	*F*(4,126) = 0.12, *p*= 0.98, $\eta ^{2}= 0.004$
Number of letters	*F*(2,126) = 2.01, $p = 0.14$, $\eta ^{2}$ = 0.03	${{\varvec{F}}}{} \mathbf (2{,}126) = \mathbf{3.48 }$, ${{\varvec{p}}} = \mathbf{0.034 }$, ${\varvec{\eta }}^{2}= \mathbf{0.052 }$	*F*(4,126) = 0.82, *p* = 0.52, $\eta ^{2} = 0.025$
Frequency (LN transformation)	$F(2{,}126) = 2.3$, $p = 0.11$, $\eta ^{2} = 0.04$	*F*(2,126) = 1.0, *p* = 0.37, $\eta ^{2} = 0.016$	*F*(4,126) = .44, *p* = 0.78, $\eta ^{2}= 0.014$

Expected main effects of valence for valence ratings and of origin for origin ratings are presented in bold. Lack of effects for all of the other controlled dimensions suggests validity of the material used

**Table 2:** Descriptive statistics (M, SD) for groups of words used in factorial manipulation

Origin category	Valence category	Negative		Neutral		Positive		Total	
	Dimensions of ANPW	*M*	*(SD)*	*M*	*(SD)*	*M*	*(SD)*	*M*	*(SD)*
Automatic	Valence	3.50	(0.36)	5.02	(0.56)	6.71	(0.35)	5.07	(1.39)
	Origin	4.45	(0.53)	4.58	(0.37)	4.33	(0.70)	4.45	(0.55)
	Arousal	4.37	(0.49)	4.15	(0.55)	4.28	(0.80)	4.27	(0.62)
	Concreteness	4.31	(1.15)	3.95	(0.74)	4.48	(1.20)	4.24	(1.05)
	NoL	7.20	(2.65)	7.47	(1.96)	7.40	(2.41)	7.36	(2.31)
	Ln_freq	5.21	(1.91)	5.65	(2.03)	5.73	(2.28)	5.53	(2.04)
Control (0)	Valence	3.37	(0.36)	5.19	(0.54)	6.38	(0.32)	4.98	(1.32)
	Origin	5.41	(0.31)	5.49	(0.30)	5.36	(0.35)	5.42	(0.32)
	Arousal	4.15	(0.23)	4.12	(0.67)	4.04	(0.51)	4.11	(0.49)
	Concreteness	4.05	(1.12)	3.96	(1.32)	4.17	(0.74)	4.06	(1.06)
	NoL	6.47	(2.03)	5.27	(1.33)	6.93	(2.02)	6.22	(1.92)
	Ln_freq	5.48	(2.28)	5.97	(1.27)	6.61	(2.02)	6.02	(1.92)
Reflective	Valence	3.66	(0.35)	5.30	(0.39)	6.49	(0.40)	5.15	(1.23)
	Origin	6.46	(0.30)	6.63	(0.41)	6.63	(0.56)	6.57	(0.43)
	Arousal	4.32	(0.49)	3.93	(0.47)	4.03	(0.36)	4.10	(0.46)
	Concreteness	4.17	(1.13)	4.09	(1.17)	4.41	(1.07)	4.22	(1.11)
	NoL	7.07	(1.75)	6.27	(1.62)	7.20	(2.27)	6.84	(1.91)
	Ln_freq	5.42	(1.37)	6.53	(1.79)	6.01	(1.22)	5.99	(1.52)
Total	Valence	3.51	(0.37)	5.17	(0.50)	6.53	(0.38)	5.07	(1.31)
	Origin	5.44	(0.92)	5.57	(0.92)	5.44	(1.09)	5.48	(0.97)
	Arousal	4.28	(0.42)	4.07	(0.56)	4.12	(0.58)	4.16	(0.53)
	Concreteness	4.18	(1.11)	4.00	(1.08)	4.35	(1.01)	4.18	(1.07)
	NoL	6.91	(2.15)	6.33	(1.86)	7.18	(2.20)	6.81	(2.09)
	Ln_freq	5.37	(1.85)	6.05	(1.72)	6.12	(1.89)	5.85	(1.84)

As demonstrated above, the intended differences can be found for valence dimension in valence groups and origin dimension in origin groups. There are no significant differences between groups for arousal, concreteness, and ln of frequency. The origin groups of words appeared to differ slightly in length. Simple contrast analysis showed that the difference concerned words of an automatic origin versus words of no particular origin: $t(132) = 2.62,\,p = 0.01$, while remaining comparisons appeared insignificant. Automatically originated words were $M = 7.3$ (*SEM* = 0.3) letters long, while words of no particular origin were $M = 6.2$ (*SEM* = 0.3) letters long.

### Warmth Versus Competence Ambiguous Task

**Fig. 2:**
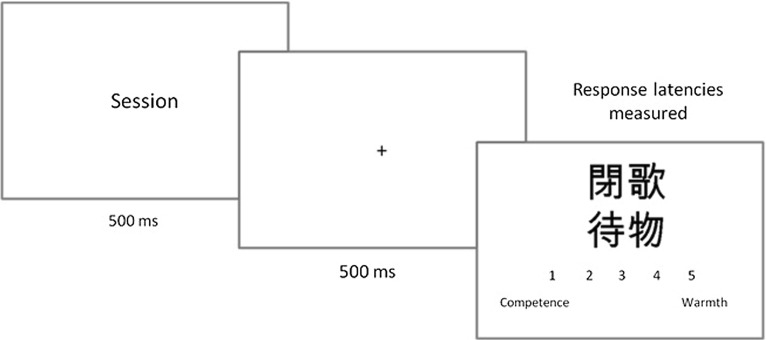
The single trial of ambiguous task composed of (1) emotional word presentation, (2) delay time for keeping the word in memory and (3) Japanese sign representing personality trait matched to competence versus warmth meaning

In order to measure the words’ meaning impact on perception of warmth and competence dimensions, an ambiguous task was prepared. The method originating from the Murphy and Zajonc (1993) paradigm was used with further modifications (cf. Blaszczak and Imbir 2012; Payne et al. 2005). The idea was simple: to present some type of unknown stimulus to participants and make them judge this stimulus in terms of warmth versus competence. The stimuli were prepared with the use of signs from the Japanese alphabet that were randomly put together in four sign compounds (cf. Fig. 2). The reason for this was to avoid symmetry differences in particular signs, by making them more complex and less symmetrical. The ambiguous task designed for this experiment was based on an assumption—expressed to participants as the truth—that the presented signs represented traits of human character or personality in the Japanese language. The task for the participants was to guess by intuition if the traits expressed by certain signs were connected more to the category of competence or warmth. The meanings of both categories were explained and definitions were provided, thus participants knew what the scale represented. Competence was defined as describing skills and abilities. Features related to competence included the ability to better solve problems and achieve better results. Competent people were described as smarter than others and able to rule others. Warmth was defined as the preferred dimension for relationships. Features related to warmth included better interaction with others or within a team. Warm people were described as having better relationships, being more popular, and attracting other people to themselves. The type of answer and reaction latencies for guessing the meaning of Japanese letters and signs were measured in this version of ambiguous task.

### Design and Apparatus

A 3 (valence) $\times$ 3 (origin) within-subject design was used. The dependent variables were (a) the type of answer given in the matching task of Japanese compound signs, and (b) the time spent on the ideogram, matching to the competence–warmth scale. The whole experiment was fully randomized. Words and ideograms appeared in a random fashion. A standard 15-in. laptop computer was used in order to present the experimental procedure to the participants. The session was prepared with E-Prime 2.0 software.

### Procedure

The study was conducted in a laboratory. Participants were invited to a study focusing on the limits of intuition and memory of briefly presented words. They were told that during experiment there would be two consecutive tasks. The first task focused on memory and involved reading words presented for half a second and then trying to remember them in the next half of a second. The difficulty of this task was explained and the participants were encouraged not to worry about the number of remembered words.

The second task was described as measuring intuition. Participants were told to look at Japanese signs describing personality traits and guess, basing on their intuition or first impressions, if the sign was connected more to the category of competence or warmth.

Participants sat in front of a computer screen and then the experiment started. During the procedure, participants were asked to try to remember as many words as possible from the 270 words presented to them. Those words were chosen in order to manipulate valence and origin (135 words as previously discussed in this manuscript) and the activation properties of words (another 135 nouns as previously planned to be discussed in another publication (c.f. Imbir 2017a). Each of the two 135 word lists appeared in separate blocks and were displayed in a random order, thus no interaction between them was expected. Also, activation words were associated with different types of signs for evaluation (i.e. hexagrams, cf. Blaszczak and Imbir 2012), thus the habituation to Japanese signs was minimized.

The single trial of this ambiguous task was composed of three stages: (1) presentation of word stimuli lasting 500 ms, (2) fixation point lasting 500 ms and (3) Japanese sign presentation with the answering scale. The idea was to briefly present a stimulus, make participants keep this stimulus in mind for a while in their working memory, and then present the intuition-based guessing probe. The words and Japanese signs were not connected explicitly in the instruction. Words were introduced as an additional task in which participants were asked to try to remember as many as possible of the words presented during the whole experiment. Figure 2 presents the single trial of the ambiguous task used in the current experiment.

After each experimental session, the direct associations of words with warmth-competence scale were assessed for each participant with use of scale from main part of experiment. “Appendix” presents values of direct associations for each word.

After the computerized section of the task, participants were given a brief memory test. Then, the actual aim of the study was expressed to them and all their questions were answered. None of the participants expected or realized during the procedure that the study was focused on the affective meaning of words. All of them reported treating both task as separate during the procedure, thus data from all samples were included in the analysis.

### Data Treatment and Analytic Strategy

A total number of 8100 trials from all 60 subjects were included in the initial analysis. The first step was to inspect reaction latencies. The initial mean of reaction latencies was $M = 2932$ ms (*SD* $=$ 4116 ms), and ranged from 9 to 76,012 ms. Outliers—reaction times lower than 350 ms (510 trials, set as a reasonable time needed to respond consciously) or longer than 13,300 ms (167 trials, set as 2.5 SD above mean)—were excluded from further consideration. Reaction times for the remaining trials were transformed by *ln*, then all data (responses, reaction latencies, and log transformed reaction latencies) were aggregated across participants and conditions. Logarithm natural transformation is a standard procedure for reaction time data, allowing the analysis of right-skewed distribution and using parametric statistics (cf., Heathcote et al. 1991).

Data was analyzed with the use of 3 (word emotional valence) $\times$ 3 (word emotional origin) within-subject ANOVA with repeated measures. The dependent variables were the types of answer chosen and log transformed reaction latency, both measured in the Ideogram warmth-competence meaning ambiguous guessing task.

## Results

### Type of Answer in Ideogram Meaning Guessing Task

**Fig. 3:**
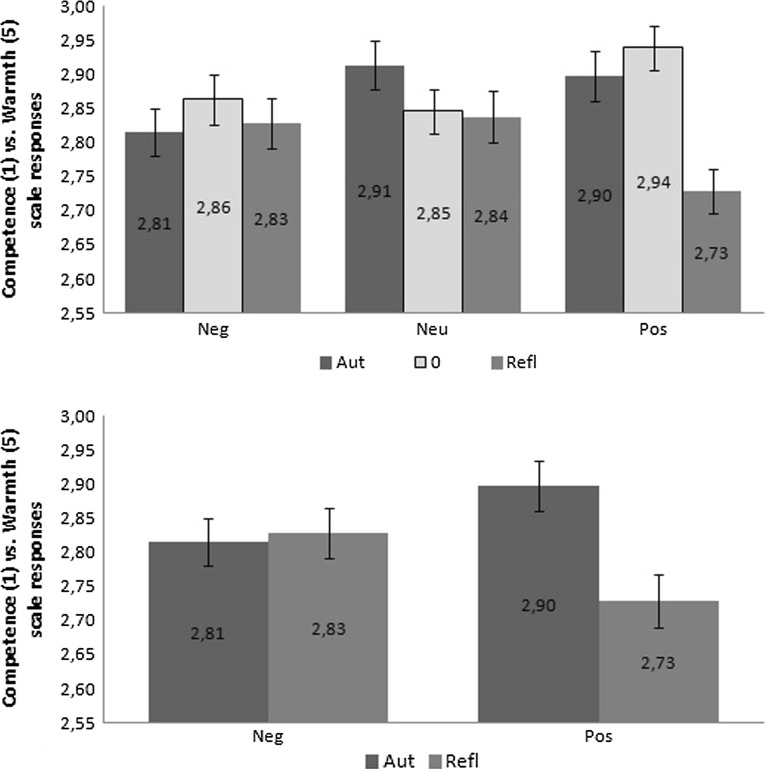
Pattern of results showing mean responses to Japanese sings in competence (1) and warmth (5) scale for all conditions (*upper plot*) and for emotive only (*lower plot*). *Error bars* represents SEM

Neither main effect of emotional valence ($F(2{,}58) = .28,\;p = .76,\;\eta ^{2} = 0.01$), nor main effect of emotional origin ($F(2{,}58) = 2.61$, $p = .08$, $\eta ^{2} = 0.08$) were found for responses in the warmth-competence dimension. Significant interaction between valence and origin of words used was found: $F(4{,}56) = 3.31$, $p = .02$, $\eta ^{2} = 0.19$. The pattern of results is shown on the upper panel of Fig. 3.

To understand the pattern of interaction found in this experiment, an additional analysis was applied to purely experimental groups of stimuli (of extreme valence and origin values) in a 2 (valence) $\times$ 2 (origin) ANOVA design. The pattern of differences remained the same. Neither main effect of emotional valence ($F(1{,}59) = .3$, $p = .86$, $\eta ^{2} = 0.001$), nor main effect of emotional origin ($F(1{,}59) = 2.88$, $p = .1$, $\eta ^{2} = 0.05$) were found for responses in the warmth-competence dimension. Significant interaction between valence and origin of words used was found: $F(1{,}59) = 5.52$, $p = .02$, $\eta ^{2} = 0.09$. Simple effect analysis showed that only statistically significant differences concerned positive words of automatic ($M = 2.9$, *SD* $=$ .57) versus reflective ($M = 2.73$, *SD* $=$ .51) origins: $t(59) = 2.78$, $p = .007$. Other possible simple contrasts remained insignificant, respectively: negative words of automatic versus reflective origins ($t(59) = -.23$, $p = .8$); automatic originated words of negative versus positive valence ($t(59) = -1.21$, $p = .23$); and reflective originated words of negative versus positive valence: ($t(59) = 1.83$, $p = .07$).

### Reaction Latencies in Ideogram Meaning Guessing Task

Neither main effect of emotional valence ($F(2{,}58) = .35$, $p = .71$, $\eta ^{2} = 0.01$), nor main effect of emotional origin ($F(2{,}58) = 1.47$, $p = .24$, $\eta ^{2} = 0.05$), were found for responses in the warmth-competence dimension. Also no significant interaction between valence and origin of words used was found: $F(4{,}56) = .31$, $p = .87$, $\eta ^{2} = 0.02$. Mean reaction time to the task was $M = 2264\,\hbox {ms}$, *SEM* $=$ 157 ms (respectively $M = 7.38$, *SEM* $=$ .065 after log transformation).

### Direct Associations Between Words Meaning and Warmth-Competence

In order to check the explanation of results in terms of semantic priming, the meaning of words direct associations with competence versus warmth 5 point Likert scale were analyzed with use of ANOVA. Assessments were provided by each participant after main experiment for each of word used. Results showed a statistically significant main effect of valence level: $F(2{,}126) = 17.03$, $p = .001$, $\eta ^{2} = 0.21$. Simple contrast analysis showed that negative ($M = 2.78$, *SD* = .57, $p = .001$) and neutral words ($M = 2.83$, *SD* = .58, $p = .001$) were perceived as more competence related than positively valenced words ($M = 3.22$, *SD* = .83) perceived as more warmth related. Also statistically significant main effect of origin level was found: $F(2{,}126) = 127.81$, $p = .001$, $\eta ^{2} = 0.67$ for direct associations in warmth-competence. Simple contrast analysis showed that automatic originated stimuli were perceived as associated more with warmth ($M = 3.54$, *SD* = .49) than neutral in origin dimension ($M = 3.07$, *SD* = .4, $p = .001$) and reflective originated stimuli ($M = 2.24$, *SD* = .42, $p = .001$). What is more reflective originated words appeared to be associated more with competence than words neutral in origin dimension ($p = .001$). Finally, there was no statistically significant interaction for direct associations in warmth-competence between valence and origin levels of words used: $F(4{,}126) = 2,3$, $p = .06$, $\eta ^{2} = 0.07$.

## Discussion

This study showed that social perception in terms of competence versus warmth is sensitive to affective manipulation provided by an unrelated task. An interaction between emotional valence and the origin of an emotional state was found. Closer inspection of this interaction revealed that the origin of affective state evoked by words alters perception in positive valence conditions, but not in negative valence conditions (cf. lower panel of Fig. 3). This is an interesting finding, showing that only positive valenced stimuli are given space in the mind to alter the perception of external objects. This is not surprising when the evolutionary perspective is considered (cf. Tooby and Cosmides 1990). It is probable that negativity is more important from a biological point of view (Cacioppo and Gardner 1999), signalizing that the organism is in danger, thus careful inspection of the object is needed. A positive state gives the subject a sense of safety and thus there is no need of careful inspection of environment and wasting energy on effortful processing. This creates a chance to use heuristically-based and simplified processing (Kahneman 2011) with employment of stereotypical categories identified in human social perception (Wojciszke 2005), that influence decisions of individuals.

The procedure used in the current study involved incidental affect (cf. Ferguson et al. 2005; Garg et al. 2005, 2007; Kenworthy et al. 2003) elicitation by word presentation. This type of procedure is based on the assumption expressed in the Russell (2003) core affect model proposition and verified later in experimental studies (e.g. Ferguson et al. 2005; Imbir et al. 2016; Imbir 2017b), that determined that simply watching affective charged materials can alter the individual’s current state of mind. This alternation causes behavioral changes in a variety of aspects, from food intake (Garg et al. 2007), categorizations (Kenworthy et al. 2003), information acquisition (Lee and Sternthal 1999) to social perception (Ferguson et al. 2005). The present study is in line with previous findings. Although there was no earlier attempt to examine the role of origin of an affective state, the current study shows that inclusion of the origin dimension in experimental design may reveal interesting effects. In fact, the valence and origin levels of materials interacted with by subjects can cause changes in a way that an ambiguous task is perceived.

The ambiguity used in the study was based on stimuli which were unknown to the participants in the form of Japanese letters compounds and the task associated with them. These, in turn, were based on the Murphy and Zajonc (1993) idea to ask participants about their suppositions about the meaning of stimuli that are meaningless to them (Blaszczak and Imbir 2012). Affective priming paradigm studies showed that people are able to process affective information presented to them in a very short exposition. But priming effect is not limited to suboptimal stimuli. The recently discovered so-called “Macbeth effect” (Zhong and Liljenquist 2006) showed that for particular cases, the priming may even be cross-modal: thinking about immoral behavior primes a behavioral tendency to clean your hands. Taking this into account, it is not surprising that a factor such as origin of emotional state may promote different ways of perceiving ambiguous stimuli. Origin of an affective state is a dichotomy proposed by Jarymowicz and Imbir (2015) to describe the duality-of-mind representation of emotional processes. Imbir (2015) proposed a continuous scale, allowing the measurement of subjective perception of the quality of people’s feelings. The origin scale represents the theoretically postulated complexity of emotional states. This complexity is distinct from purely cognitive complexity measured in the dimension of concreteness (cf. Imbir 2016b). Correlational analyses of origin and concreteness showed that they share no more than 10% of common variance, thus may be treated as distinct constructs. In the current study, concreteness was aligned across conditions, thus the effects observed may be attributed only to the emotional complexity of underlying mechanisms for automatic and reflective origins.

In this study, automatic originated stimuli were expected to promote the processing of ambiguous Japanese signs in terms of warmth, due to sharing by both automatic evaluative system and warmth dimension the primacy in mind processing (cf. Jarymowicz and Imbir 2015; Kahneman 2011; Willis and Todorov 2006; Ybarra et al. 2001). Reflective originated stimuli were expected to be associated with perception in terms of competence, due to the effort associated with more complex emotional processes (Jarymowicz and Imbir 2015). Direct associations measured for words used in experimental procedure, assessed by subjects participating in the study, showed that indeed automatic originated stimuli evoked warmth associations while reflective originated stimuli evoked competence related associations. Origin related result for positive valenced stimuli are consistent with the claim that Affect Misattribution Procedure is more sensitive to semantic rather than affective aspect of priming stimuli (Blaison et al. 2012). In the case of current study, no valence effects were found, but only for positive valenced stimuli, the expected priming results were found. It is worth to highlight that valence results did not follow the pattern suggested by semantic priming. There were no valence specific effects, this suggests that the hypotheses made are valid.

### Limitations and Conclusions

The current study has some limitations. In this study, the warmth and competence dimensions were utilized on a single scale, while in social perception they are considered as distinct constructs (Fiske et al. 2007; Wojciszke 2005). The rationale for this is that at the initial stage of investigations, the proposed method allowed the identification of the main tendencies in perception. Also, this allowed the measurement of both dimensions in a single experiment, which is important when seeking to find the dominant tendency. Similar problems can be found in the negative-positive dimensions used in the description of bad versus good objects in affective priming paradigm studies (Murphy and Zajonc 1993). Negativity and positivity can be treated as single bipolar dimension or two distinct constructs (cf. Cacioppo and Gardner 1999), each approach giving fruitful outcomes. Still, warmth and competence are distinct constructs and dimensions, but only used here on a single scale.

Another issue worth discussing is the validity of stimuli used in the current experiment limited to the certain population involved in normative studies for words (Imbir 2015, 2016b). In other words, although origin of an affective reaction concept is a rather universal factor and should be present in all cultures (c.f. Imbir 2015, 2016a; Jarymowicz and Imbir 2015), the perception of what is automatic and reflective in origin, operationalized and measured in normative studies for words (c.f. Imbir 2016b), may be specific to culture or age group of participants, therefore, stimuli presented in “Appendix” are valid only for a specific population (namely Polish students aged from 18 to 30). From that reason, the replication of this study require: (1) reassessment of origin of an affective state with use of SAM scale for Origin (Imbir 2015) in another culture or age group, then (2) stimuli selection based on a procedure analogical to this one described in the method section.

In conclusion, it is worth highlighting that the present study, with the use of precisely selected verbal stimuli, showed that the meaning of ambiguous objects can be primed by the affective quality of those stimuli. Results suggest that the duality-of-mind framework applied to diverse emotional states allows us to find the link between incidental affect and social perception. Only positive valence promoted the use of stereotypical knowledge of warmth and competence, which was primed by the origin of verbal stimuli used for the task of guessing the meaning of unknown stimuli.

## Appendix 1

**Table:**

Polish word	English translation	Category	Valence category	Origin category	Val *M*	Origin *M*	Arousal *M*	Concreteness *M*	Freq	NoL	Competence-warmth direct associations
czkawka	Hiccup	ANeg	1	1	4.04	4.54	3.86	3.18	79	7	3.22
szloch	Sob	ANeg	1	1	3.04	3.58	4.70	3.68	735	6	3.81
łzy	Tears	ANeg	1	1	3.66	3.38	4.54	2.08	660	3	3.97
uszczypnięcie	Pinch	ANeg	1	1	4.06	4.38	4.38	3.44	24	13	3.42
pijak	Drunk	ANeg	1	1	2.76	4.98	5.40	2.84	467	5	3.03
naiwniak	Sucker	ANeg	1	1	3.38	4.16	3.84	5.40	23	8	3.15
słabeusz	Weakling	ANeg	1	1	3.18	4.98	3.58	5.39	32	8	2.95
zmęczenie	Fatigue	ANeg	1	1	3.28	4.90	3.50	5.56	2044	9	3.44
hałas	Noise	ANeg	1	1	3.56	4.74	4.60	4.18	3199	5	2.86
plotka	Rumor	ANeg	1	1	3.50	4.78	4.48	5.04	588	6	3.71
grymas	Grimace	ANeg	1	1	3.76	4.42	4.52	4.20	1618	6	3.36
gafa	Blunder	ANeg	1	1	3.66	4.42	4.58	5.18	23	4	3.15
usidlenie	Ensnaring	ANeg	1	1	3.88	4.90	4.52	5.38	9	9	2.98
smarkacz	Stripling	ANeg	1	1	3.36	4.88	4.64	3.46	265	8	3.59
zaślepienie	Infatuation	ANeg	1	1	3.32	3.66	4.40	5.64	75	11	3.36
procesja	Procession	ANeu	2	1	4.76	4.88	3.50	3.56	293	8	2.95
kościół	Church	ANeu	2	1	5.24	4.46	3.54	3.78	3652	7	3.14
kuksaniec	Nudge	ANeu	2	1	4.53	4.55	4.10	3.02	13	9	3.1
tarot	Tarot	ANeu	2	1	4.16	4.90	3.72	3.92	38	5	2.97
loteria	Lottery	ANeu	2	1	5.76	4.70	4.10	3.46	56	7	2.75
westchnienie	Sigh	ANeu	2	1	5.48	4.28	3.60	4.44	1336	12	3.98
jałmużna	Alms	ANeu	2	1	4.36	4.84	4.04	3.92	44	8	3.2
błazen	Clown	ANeu	2	1	4.64	4.62	4.28	3.26	507	6	3.08
mrowienie	Tingling	ANeu	2	1	4.26	4.90	3.96	4.39	482	9	3.42
pragnienie	Desire	ANeu	2	1	5.14	3.40	5.18	5.80	4066	10	3.81
obrzęd	Rite	ANeu	2	1	5.04	4.76	3.66	4.90	220	6	3.12
wróżka	Fairy	ANeu	2	1	5.60	4.68	4.18	4.30	338	6	3.41
młodzież	Youth	ANeu	2	1	5.68	4.50	4.66	3.40	1703	8	3.59
łasuch	Gourmand	ANeu	2	1	5.74	4.72	4.40	4.00	9	6	3.51
burza	Storm	ANeu	2	1	4.86	4.54	5.30	3.06	3238	5	3.53
zakochanie	Infatuation	APos	3	1	7.56	2.28	6.50	7.24	52	10	4.75
passa	Streak	APos	3	1	6.16	4.84	4.36	5.78	41	5	3.02
toast	Toast	APos	3	1	6.36	4.60	4.30	3.92	689	5	3.69
powitanie	Welcome	APos	3	1	6.50	4.96	3.90	4.70	1825	9	3.88
zapach	Fragrance	APos	3	1	6.80	4.66	3.70	4.28	9963	6	4
słodycz	Sweetness	APos	3	1	7.02	4.44	4.14	3.54	477	7	4.25
pomoc	Help	APos	3	1	6.84	4.48	3.54	5.06	10180	5	3.93
niemowlak	Infant	APos	3	1	6.50	3.86	3.70	2.50	28	9	4.36
flirt	Flirt	APos	3	1	6.46	3.36	5.52	5.72	146	5	4.56
potomstwo	Offspring	APos	3	1	6.62	4.60	3.68	3.36	504	9	4
pozdrowienie	Greeting	APos	3	1	6.72	4.60	3.58	5.40	364	12	4.14
skarb	Treasure	APos	3	1	6.84	4.76	4.20	3.72	2460	5	3.05
walentynka	Valentine	APos	3	1	6.42	4.26	4.66	4.24	2	10	4.31
podarunek	Gift	APos	3	1	6.78	4.44	4.30	3.56	373	9	3.98
ferie	Holiday	APos	3	1	7.10	4.82	4.12	4.14	123	5	3.63
wina	Fault	ONeg	1	2	3.46	5.18	4.52	5.78	9887	4	3.1
ciemnota	Unacquaintance	ONeg	1	2	3.16	5.38	4.34	5.41	87	8	2.85
truchło	Carcass	ONeg	1	2	3.56	5.16	3.98	2.90	68	7	2.73
dół	Pit	ONeg	1	2	4.04	5.52	4.02	3.28	24000	3	2.64
ochłap	Offal	ONeg	1	2	3.28	5.60	3.80	3.72	118	6	2.76
breja	Slush	ONeg	1	2	3.49	5.90	4.10	3.11	43	5	2.86
paszkwil	Libel	ONeg	1	2	3.57	5.48	4.51	5.38	69	8	3.02
kuternoga	Lame	ONeg	1	2	3.77	5.07	4.13	2.96	37	9	2.97
reumatyzm	Rheumatism	ONeg	1	2	2.96	5.90	3.72	3.96	211	9	3.02
biedak	Wretch	ONeg	1	2	3.48	5.18	4.16	3.22	794	6	3
śpiączka	Coma	ONeg	1	2	2.44	5.36	4.02	3.70	53	8	2.93
obtarcie	Sore	ONeg	1	2	3.46	5.06	4.22	3.20	7	8	2.92
błąd	Error	ONeg	1	2	3.38	5.46	4.34	5.68	5631	4	2.15
łachmany	Rags	ONeg	1	2	3.26	4.98	4.30	3.04	387	8	2.71
wada	Drawback	ONeg	1	2	3.30	5.86	4.12	5.36	211	4	2.78
doping	Cheering	ONeu	2	2	5.24	5.12	5.18	4.46	23	6	2.81
chór	Choir	ONeu	2	2	5.71	5.40	3.54	2.78	1267	4	3.05
kłębek	Hank	ONeu	2	2	5.40	5.72	3.58	2.68	1093	6	2.92
telewizja	Television	ONeu	2	2	5.24	5.74	3.54	3.14	529	9	2.86
guru	Guru	ONeu	2	2	4.94	6.04	4.00	5.56	199	4	2.63
wódka	Vodka	ONeu	2	2	5.60	5.10	5.76	1.58	568	5	3.34
unik	Dodge	ONeu	2	2	4.96	5.48	4.42	4.38	461	4	2.54
czara	Goblet	ONeu	2	2	5.48	5.43	3.68	2.65	214	5	3.1
smok	Dragon	ONeu	2	2	5.66	5.14	4.58	4.18	3438	4	3.36
blef	Bluff	ONeu	2	2	4.10	5.52	4.28	5.64	136	4	2.92
żargon	Jargon	ONeu	2	2	4.94	5.65	3.67	5.02	174	6	2.98
głębia	Depth	ONeu	2	2	5.26	5.38	3.74	5.48	243	6	3.5
farsa	Farce	ONeu	2	2	3.98	5.08	4.54	5.54	144	5	2.85
grono	Bunch	ONeu	2	2	5.60	5.70	3.60	3.30	574	5	3.81
pisarz	Writer	ONeu	2	2	5.74	5.92	3.76	2.98	2412	6	2.85
przyjęcie	Party	OPos	3	2	6.78	4.96	4.54	3.88	3452	9	3.95
rejs	Cruise	OPos	3	2	6.44	5.40	3.64	3.06	689	4	3.08
powiew	Waft	OPos	3	2	6.10	5.45	3.50	3.66	1391	6	3.61
promocja	Promotion	OPos	3	2	6.70	5.68	4.56	4.68	61	8	2.83
klimat	Climate	OPos	3	2	6.06	5.86	3.86	3.90	824	6	3.61
gość	Guest	OPos	3	2	6.42	5.38	3.76	3.28	4380	4	3.47
brawa	Applause	OPos	3	2	6.68	4.76	4.80	4.40	1053	5	3.42
kreskówka	Cartoon	OPos	3	2	6.82	5.14	3.96	3.04	2	9	3.44
melodia	Melody	OPos	3	2	6.88	5.24	3.46	4.50	750	7	4.12
wydarzenie	Event	OPos	3	2	5.86	5.90	4.54	4.52	1417	10	2.76
smak	Taste	OPos	3	2	6.32	5.04	3.56	4.54	2946	4	3.53
południe	South	OPos	3	2	6.04	5.10	3.58	4.24	9135	8	3.53
malarstwo	Painting	OPos	3	2	6.12	5.24	3.58	4.38	321	9	3.29
wyzwanie	Challenge	OPos	3	2	6.24	5.34	4.66	5.92	1521	8	2.54
obrońca	Defender	OPos	3	2	6.30	5.88	4.64	4.49	564	7	2.93
egzaminy	Exams	RNeg	1	3	3.60	7.02	5.60	3.84	340	8	1.75
ignorancja	Ignorance	RNeg	1	3	2.98	6.14	4.68	6.44	140	10	3.02
krata	Grating	RNeg	1	3	3.98	6.16	3.60	1.74	357	5	2.42
minus	Minus	RNeg	1	3	3.84	6.36	3.52	4.90	554	5	2.17
szpieg	Spy	RNeg	1	3	3.92	6.72	4.20	3.30	637	6	1.9
koszty	Costs	RNeg	1	3	3.78	6.40	4.08	3.88	1134	6	1.92
podwładny	Subordinate	RNeg	1	3	4.12	6.28	4.02	4.06	189	9	2.17
podatek	Tax	RNeg	1	3	3.32	6.92	4.22	3.60	228	7	1.85
alimenty	Alimony	RNeg	1	3	3.60	6.34	4.48	3.42	82	8	2.31
odsetki	Interest	RNeg	1	3	3.78	6.56	4.36	3.80	58	7	1.98
rząd	Government	RNeg	1	3	3.80	6.50	4.50	3.64	5596	4	1.83
przemyt	Smuggling	RNeg	1	3	3.70	6.68	4.60	4.00	180	7	2.31
recesja	Recession	RNeg	1	3	3.63	6.65	4.20	5.13	25	7	2.03
bezrobocie	Unemployment	RNeg	1	3	2.92	6.06	4.20	5.40	122	10	2.1
heretyk	Heretic	RNeg	1	3	3.94	6.10	4.58	5.42	45	7	3.07
szlachta	Nobility	RNeu	2	3	5.46	6.22	4.08	3.90	811	8	2.31
etykieta	Label	RNeu	2	3	4.90	6.60	3.52	3.08	126	8	2.49
sułtan	Sultan	RNeu	2	3	5.10	6.84	3.46	3.10	397	6	2.31
zadatki	Makings	RNeu	2	3	5.32	5.98	3.80	5.34	94	7	2.36
prawo	Right	RNeu	2	3	5.84	7.60	3.68	5.96	19169	5	2.07
prasa	Press	RNeu	2	3	5.30	6.64	3.50	2.84	1232	5	2.61
stawka	Bid	RNeu	2	3	5.42	6.28	4.42	4.02	301	6	2.05
raport	Report	RNeu	2	3	4.88	6.98	3.56	2.84	2634	6	1.97
wojsko	Army	RNeu	2	3	4.94	6.62	4.96	2.90	2893	6	1.88
interes	Business	RNeu	2	3	5.86	7.10	4.36	4.82	3421	7	1.79
dyscyplina	Discipline	RNeu	2	3	5.46	6.44	3.96	5.74	384	10	1.92
wynik	Result	RNeu	2	3	5.52	6.70	4.60	4.58	1919	5	2.14
weto	Veto	RNeu	2	3	4.41	6.62	4.02	5.57	19	4	2.15
hodowla	Breeding	RNeu	2	3	5.56	6.12	3.62	2.82	131	7	2.63
kurs	Course	RNeu	2	3	5.48	6.66	3.48	3.82	2801	4	1.98
miliard	Billion	RPos	3	3	7.08	7.06	4.68	4.18	311	7	2.12
tolerancja	Tolerance	RPos	3	3	6.62	5.32	3.88	7.32	139	10	3.71
mistrz	Master	RPos	3	3	7.22	6.24	4.52	4.38	4209	6	2.37
patent	Patent	RPos	3	3	5.92	7.59	3.52	4.62	221	6	2.07
dobytek	Property	RPos	3	3	6.48	6.76	3.74	3.78	552	7	2.49
absolwent	Graduate	RPos	3	3	6.38	6.56	3.90	3.44	206	9	2.39
uczony	Scholar	RPos	3	3	6.26	7.44	3.58	5.04	1421	6	1.71
stypendium	Scholarship	RPos	3	3	7.06	6.62	4.12	3.16	372	10	1.83
szczyt	Peak	RPos	3	3	6.44	6.12	4.08	3.28	3533	6	2.39
równowaga	Balance	RPos	3	3	6.08	6.32	3.56	5.38	367	9	2.73
oszczędności	Savings	RPos	3	3	6.68	6.94	3.94	4.40	737	12	2.05
płaca	Wages	RPos	3	3	6.16	6.82	4.27	3.27	63	5	1.9
satyra	Satire	RPos	3	3	6.04	6.16	4.04	5.10	171	6	3.41
lider	Leader	RPos	3	3	6.22	6.58	4.50	4.06	90	5	2.15
zysk	Profit	RPos	3	3	6.78	6.88	4.18	4.76	651	4	2.05
